# Artificial Humic Acids: Sustainable Materials against Climate Change

**DOI:** 10.1002/advs.201902992

**Published:** 2020-01-21

**Authors:** Fan Yang, Markus Antonietti

**Affiliations:** ^1^ School of Water Conservancy and Civil Engineering Northeast Agricultural University Harbin 150030 China; ^2^ Joint laboratory of Northeast Agricultural University and Max Planck Institute of Colloids and Interfaces (NEAU‐MPICI) Harbin 150030 China; ^3^ Max Planck Institute of Colloids and Interfaces Department of Colloid Chemistry 14476 Potsdam Germany

**Keywords:** CO_2_ remediation, humic acid, hydrothermal processes

## Abstract

Humic acid, as a natural organic matter, is widely distributed in surface soil, oceans, rivers, and other ecological environments throughout the whole earth ecosystem. Humic acid provides abundant organic carbon and helps to maintain a hydrated, pH and redox buffered environment hosting the soil microbiome. Humic acid is however also a largely ignored polymer material full of exciting functional properties, and its scale is enormous. This perspective article discusses its synthesis and management as a tool to tackle parts of the climate crisis as well its use in technological applications, as made by chemical conversion of agricultural side products to artificial humic acids.

## Graphical abstract

Carbon bound to the topsoil as humic matter exceeds by overall mass the carbon of biosphere as well as in the atmosphere. While binding CO_2_ in biomass is a subsidized tool, binding CO_2_ in fertile soils is rarely done, despite its breath‐taking potential. Herein, the “artificial humification” to shift 200 Gt of carbon from the atmosphere to soil is discussed.

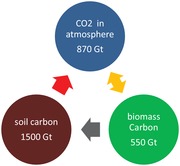

## 1 Introduction

The organic matter fraction in the geological carbon pool is unimaginably big: it contains an estimated 10^16^ tons of carbon, exceeding the total organic content of current living matter by a factor of 10 000.^1^ Even restricting counting to the humin/humic acid fraction in the upper layers of farmed soil, the numbers are very high, and an overall of 1500 × 10^9^ t is reported.^2^ Soil thereby contains by far more carbon than the atmosphere (currently 870 × 10^9^ t,^3^), it is the most important player in the global carbon cycle,^4^ literally mostly unseen by the public. CN&E in a cover story nicely compacted the whole context in a single quote by Debbie Barker (the International Director of the Center of Food Savety): “We have a huge potential carbon sink below our feet, and we are not taking advantage of it.” We recommend the original article for more details). Soil organic matter is in major parts a long‐lasting, accumulated product of the degradation of land plants, and a change of its content in the range on larger soil areas has the potential to cure the climate crisis completely. For that, the so‐called 4 per 1000 initiative claims that an increase by 0.4% of soil carbon in farmed areas every year is enough,^2^, ^5^ and considering on top the large contribution of nonfertile, carbon poor badlands, this is not difficult at all.

As another important fact for discussion is, photosynthesis of land plants immobilizes more than 220 billion tons of CO_2_ per year globally,^6^ these correspond to an uptake of 27.8 permille atmospheric CO_2_ per year in absolute units (this taken as the maximal theoretical recovery rate based on terrestrial plants). As a Gedankenexperiment, the atmospheric CO_2_ could be “eaten up” to pre‐industrial levels in about 5 years, only. But: without interference only a small amount of this immobilized carbon ends up throughout natural processes in soil carbon, as dead plant matter is mostly metabolized (to methane and CO_2_), with little remainders left to form the soil carbon pool.

**Figure 1:**
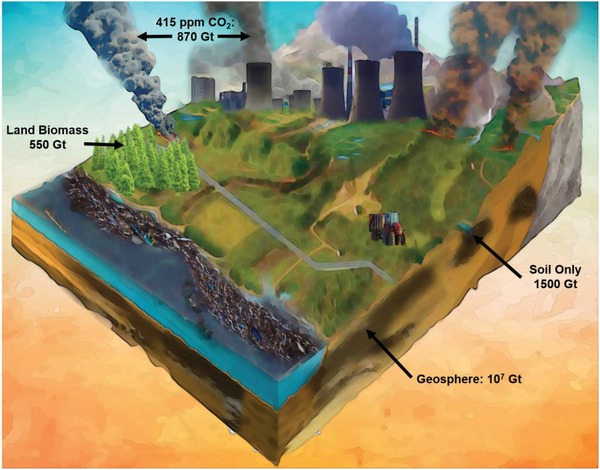
Comparison of different carbon pools to illustrate relative importance.

Talking about soil carbon as a chemical product of tool thereby has a significant scale and importance: is by far larger than all human chemical activities in energy and chemistry, just that we do not actively do it. **Figure** 1 compares the different carbon pools also graphically to illustrate relative importance and size.

From a viewpoint of a carbon scientist who has specialized on carbons and coals, there is obviously a big confusion on what soil carbon is. There is certainly a particulate fraction which is a solid with a C‐content higher than 70 wt% which cannot be dissolved or swollen, and therefore exists only as a particle or porous scaffolds. The product is highly condensed by biological processes or fire. The notation “Biochar,” i.e., flame carbonized lignocellulosic biomass, refers to the trials to copy this type of soil carbon, and is applied in farming experiments in a variety of fashions (to be put into the superficial, plough and underlying layer of soil). We meanwhile think that this is too simple, and the following discussion is not on the “hard” part of the soil carbon. In fertile soils or in peat, the carbons are less condensed, and at least according to our experiments, a big majority of carbon is extractable and thereby humic/humic acid in nature. The notation “humic acid” (HA) comes from the early chemical experience that strong bases dissolve significant parts of the black‐brown material, while re‐acidification results in precipitation. There are also minor parts of humic matter become swollen but stay undissolved, but as its chemistry and composition is very similar, we can simplistically assume that this is only the more hydrophobic or cross‐linked part of the same, broad polymer material.

Without doubt, humic matter is well‐explored in geochemistry and soil science, but a pure chemical lab‐synthesis of HAs, their chemical modification, and their valorization as an advanced polymer material is largely missing. The wish to create a sustainable chemistry and to tackle the climate crisis actively will however move HAs out of the classical soil context and to applications in chemical technology and for remediation. This is what this perspective article is about.

What is this HA and how to make it, best without losing any carbon in metabolization? HA as extracted by standard protocols from soil is a complex polymer compound that contains acidic carboxyl and phenolate groups. Just recently, modern analytic techniques have improved our understanding of the inner molecular construction principles. Here, advanced solid‐state NMR,^7^, ^8^ but also the tools of metabolomics were applied to HA, and the resulting so‐called “Humeomics” allowed identification of many of the molecular sub‐architectures.^9^ Also by means of XPS, scientists can quantify the element composition at the surface of the solid material, and by fine structure analysis of the peaks detailed information about the binding environments of each element can be gained.^10^ This can be complemented by IR spectroscopies and pyrolysis‐mass spectrometry,^11^, ^12^ so that to our opinion meanwhile stable analytical knowledge for quantitative discussions also in a pure materials environment is reached.

**Scheme 1:**
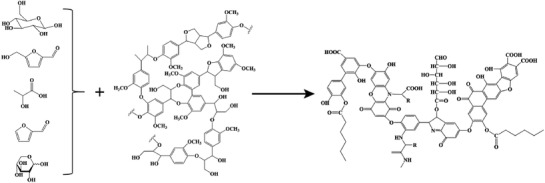
Simplified structure of an average humic acid.

The structure is thereby complex and varies in a certain range, according to the origin, but the average properties of extracted humic substances are remarkably similar and resistant to further biodegradation.^10^ The communalities are so large that we indeed speak about one class of organic, covalent compounds. HAs are random polycondensates with polyelectrolyte/polyampholite behavior with a polyphenol or quinone‐based aromatic core, in which functional side structures contain carboxylic, phenolic, and carbonyl groups, as well as sugar, peptide fragments. The subentities are connected by various connectors, such as —O—, —CH_2_—, =CH—, —NH—, —S—S—, and similar groups.^13^, ^14^ The contained chemical functionality determines its properties in ion exchange, water absorption, its dispersion and cohesion energy, as well as interactions and complexation. A scheme of the general structure is illustrated in **Scheme** 1.

## 2 From Natural Huminogenesis to Chemical Retrosynthesis

Huminogenesis describes the physical, chemical, and biological cascades toward organic humic matter, and careful observation of the natural processes allows us to identify the schemes to work on a potential “synthetic lab approach” to artificial humic acids (A‐HA). The formation of humic substances under natural conditions requires many years and involves biotic and abiotic reactions. Black soil is mostly found in zones with four distinct seasons and phases of humidity throughout the year, while the soil can be deeply frozen in winter. This causes a slower metabolization of dead biomass by microbial activity and allows chemical reactions to occur, thus resulting overall in the slow formation of a humus layer. An exciting subcase are peatlands which as carbon‐rich ecosystems store one‐third of the soil carbon but cover just three percent of the land surface of the earth.^15^ The transformation of organic matter occurs here mostly under waterlogged nonaerobic conditions with low pH and low oxygen availability.^16^ A near neutral pH with high buffering capacity is another characteristic of black soil. As a third interesting fact, organic decomposition cascades in presence of soil minerals are “self‐neutralizing,” i.e., even if we have to start at both rather acidic and alkaline starting situation, the different organic cascades generate organic counter‐products which prompt neutralization at the end. The last two processes are copied by the currently most effective chemical engineering processing toward A‐HA, the so‐called “hydrothermal humification” (HTH, see ref. 1).

In this process, wet biomass is hydrothermally treated under exclusion of oxygen and at autogenous steam pressure. HTH can be considered a comparably mild chemical engineering process simulating natural humification, but due to the significantly increased temperature, it is accelerated down to reaction times of only minutes to a few hours. In addition, as a chemical process, HTH usually achieves excellent carbon yields and metabolization is avoided, i.e., the majority of the carbon bound in biomass ends up in the humified product. The carbon yield is also much higher than in flame coalification, where a major part of the biomass carbon is oxidized and released as greenhouse gases (CO_2_ and CO).^17^, ^18^ Hydrothermal carbonization without further modifications (HTC) usually gives hydrochar,^19^ i.e., a solid, insoluble, non swellable product very similar to biochar. The base soluble character of HA is generated by a bioinspired modification. Water‐logged conversion of biomass usually starts biologically as a mixed acid fermentation by Enterbacteriaceae, i.e., swamps turn acidic. Under these conditions, in a second step slow chemical humification under acid catalyzed dehydration of sugars take places, in which the first formed organic acids are built into the HA, thus leading to the name‐giving carboxyl groups. To mimic the first step organic acid formation, hydrothermal chemistry can start the reaction with appropriately alkaline solutions which then stimulate the auto‐neutralizing formation of exactly the corresponding amount of carboxylate groups by retro‐aldol addition from the sugars.^20^ After short reaction time and practically independent of the degree of alkalinity, the created acids turn the overall reaction solution into weakly acidic; thus driving the dehydration of sugars to form furan derivates, such as hydroxymethylfurfural, which then condense together with the organic acids to the covalently humic polymer condensate.^1^ Like that, both mixed acid generation as well as humification can be successively mimicked in one single reactor, just using the auto‐neutralization principle discussed above, and with the amount of base exactly controlling the number of acid functionalities. Lignin is an important co‐reactant to bring the mandatory phenolic sites to the product, and their amount in the final A‐HA exactly follows the included lignin content.

Analyzing natural HAs, one can find fatty acids, which come from microbial lipids, as well as Amadori‐like amino acid condensates as minority costructures in the final product. For the synthetic design of specialty polymers based on HA, they however point to a possible structural diversity to increase hydrophobicity and plasticity of the material (fatty acids), or to increase nitrogen content and secondary interactions (amino acids).

## 3 Potential Applications of Artificial Humic Acids

To have access to a cheap, omni‐available, and fully sustainable natural polymer with a high extend of functionality is interesting for many applications, especially when those properties include amphiphilicity, ion binding and polyelectrolyte behavior, as well as (phenol‐based) redox properties. As A‐HA is essentially carbon negative (it binds carbon while its formation has avoided metabolization and CO_2_ generation) and it is made essentially only from side products or waste streams, application of A‐HA as a replacements of fossil products or on larger scale in agriculture can contribute to find a way to a CO_2_‐neutral society.

Agricultural usage of HA is a traditional, well developed application, and the weakly condensed precursor, compost, does not only P, N, and K and other essential elements as such, but promotes plant growth beyond that. Metabolites included in HA can serve as phytohormones,^21^ while the complexes of HA with salts, inorganic clusters and minerals bind water, retain nutrients in soils and thereby make up land quality.^22^ Addition of humic substances is now a part of modern agricultural practice as both application of irrigation and fertilizer can be lowered. The actual picture of the role of HA in soil fertility is multifacetted and includes physico‐chemical aspects as wettability and soil textural aspects as well as the increase of water and ion binding capacity. Generally, HA can exchange, adsorb and complex mineral elements, making it a good fertilizer synergist.^23^ For example, humic acid in soil is positively correlated with the mineralization rate of organic nitrogen. The addition of humic acid makes fertilizer decomposition slower and more plant effective. Moreover, it can improve the mobility of phosphorus in the soil and slows down the process of phosphorus remineralization.^24^

HA also improves soil structure,^25^ i.e., it binds dispersed soil particles together, making the soil become more likely to form agglomerates, and then the aeration, water permeability and soil water retention capacity are enhanced. HA drives microbial activity in soil, that is, increases the number of microbial communities and catabolic efficiency. Furthermore, the highest activities of soil enzyme as well as the highest functional diversity can be found soil fractions with abundant organic matters.^26^ Flaig et al.^27^ published a report on the application of HA to improve the early resistance of plants and significantly promote plant growth as early as the mid‐19th century. Although studies on the effects of humic acids on plant growth have been initiated long ago, humic acids are still far from being completely understood.

HA addition was found to be beneficial not only for improvement of still operated farmland, but also for the reconstituted or contaminated soil by physical and chemical adsorption, precipitation and redox buffering. Heavy metal pollution can be reduced by ion exchange, complexation and surface adsorption, while even persistent organic pollutants are first adsorbed, then decomposed by redox processes.^28^, ^29^, ^30^ As HA was found active in such applications already at dose levels typical for functional polymers in technical formulations, the use of a then optimized A‐HA for agriculture is a more than promising challenge.

Ion binding in HAs is special and extends ordinary ion exchange resins. The contained carboxylate groups bind the usual ions (Na^+^, K^+^, or Mg^2+^, Ca^2+^, Fe^2+^) by Coulomb interactions, while the phenol and phenolate groups are extremely efficient for metal chelation (all d‐elements, especially Fe^3+^). In addition, hydrated ions as phosphate bind via H‐bridges and surface adsorption (the “hydrophobic” effect). As that, the measured ion binding capacities and ion binding constants as well as water wettability and uptake, reflect the presence of a multiplicity of very diverse binding sites along the conjugated backbone. Ordinary peat was already employed for metal removal^31^ and was found to be a cheap and environmental benign solution. As any ion exchange resin, the peat can be reactivated by leaching the ions with acid. Optimal ion binding is obtained at slightly acidic conditions (pH = 5.5–6.5), which underlines the role of phenols, not phenolates, in this process.

In another work binding constants and binding capacities for diverse metal cations with HAs were determined^32^ and found to be rather high. The authors have said that “an organic ligand like humic acid may render almost all metals immobile, thereby helping in detoxication processes.” This was in fact repeated in many other examinations, all reporting on fast, strong, and efficient ion binding by isolated humic matter. For instance, our group^33^ synthesized a chemically modified hydrothermal carbon from glucose and acrylic acid and described Pb^2+^ binding of up to 350 mg g^−1^ at pH 6.0.

HAs can also bind organic pollutants in water via hydrophobic and secondary interactions, thus reducing volatility, increasing the photolysis rate as well as changing the biological availability and the toxicity.^34^, ^35^ Here, the aromatic core structure of humins provides the binding site of organic molecules.^36^ Toxicity of organic pollutants is lowered by the addition of HA, as binding to surfaces reduces the molecular solubility and thereby reduces bioavailability. The higher the binding constant toward the specific humic matter is, the lower is the toxicity.^37^

For us, the most surprising property of HA and A‐HA is however the redox capacity. As humic acids are largely heteroaromatic or phenol based, a very pronounced redox activity similar to semiconducting polymers is nearby to expect, and indeed, the numbers of electrons (and protons) which can be stored in humic matter are unexpectedly large. Maurer et al. have applied electrochemical reduction of extracted HAs and quantified an uptake of 0.54 mol protons kg^−1^ and 0.55 mol electrons kg^−1^.^38^ Even when compared to a specially designed, synthetic electrochemically active polymer, these values (in battery language 27.8 Ah kg^−1^) are promisingly high. In simple words: humic acid has 15% of the storage capacity of an optimized lithium ion battery material! It is a real surprise to a material chemist that such a property of black soil is not discussed in broader context and optimized for materials applications before. The parental lignin is a redox‐active compound too, and here, in optimized systems values of ≈80 Ah kg^−1^ were accessed.^39^ The redox activity of humic acids can thereby be related to the phenolic sites brought in by the lignin fraction, which makes up about 30 wt% in most humic matter. The importance of such a redox capacity in natural and artificial humic acids For instance, this is the reason why soil indeed represents stable, pH and redox buffered medium for microbial growth, comparable as externally controlled media as any bioreactor or the cellular body. A very tutorial case to illustrate the ecological importance was reported by Kappler et al.^40^ They analyzed electron shuttling via humic acids in freshwater sediments of the lake Constance and found mostly Fe(III) in the aerobic top layers, where Fe(II) was dominant in the anaerobic lower layers. When analyzing the bacterial polytype, they found substantially larger populations of humic acid reducing bacteria than those who reduce iron, with HA based bacteria being as many as the fermenting bacteria. This of course underlines the central role of redox‐active humic acids buffer sensible ecological communities.

**Figure 2:**
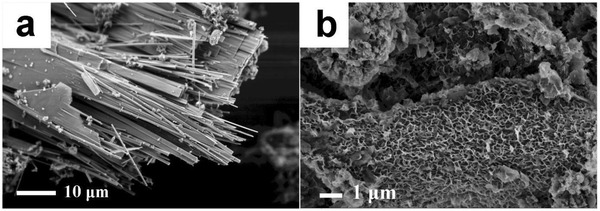
Recrystallization and morphology changes by interaction with an artificial humic acid. a) Iron phosphate nanofibers after HTH reaction, b) stabilization and tectonic rearrangement of clay nanosheets in treated waste water sludge.

Another potential megascale application of HAs and A‐HAs is based on their strong interaction with minerals. Modern investigations of clay‐straw bricks as the sustainable building material of tribal cultures have found that the included humic acids bind to the clay particles and thus increase the toughness and plasticity.^41^ Humic acids might therefore replace commercial fossil‐sourced cementum flow additives, again a globally significant potential reduction of CO_2_ footprints. Considering the very strong ion binding, humic acids should play a key role in crystal morphosynthesis in general,^42^ and it is certainly also relevant for mineralization and remineralization processes in soil. In a recent paper,^43^ we even analyzed the action of A‐HA on otherwise insoluble phosphates (taking FePO_4_ as a model), and total crystal reconstitution and highly increased bioavailability was found. **Figure** 2 shows a preliminary experiment where A‐HA was added to crystals throughout hydrothermal treatment, and a complete morphology change toward high surface area species was found.

## 4 A New Chemistry for Climate Remediation?

It was mentioned in the introduction: the carbon content of humic matter significantly exceeds all carbon contained in the biosphere and the atmosphere, so considering climate solutions with only biomass (e.g., reforestation) leaves out the biggest factor of all: the soil. As it was discussed by the “4 per 1000” initiative:^3^ a possible little increase of the humic matter concentration in soil has the potential to cure the (complete) climate problem while bringing improved agricultural fertility and food safety at the same time. The numbers are big but easy: 120 ppm CO_2_ reduction in the atmosphere, i.e., our complete debt of industrialization, is only a 16% relative increase of humic matter in soil. The “4 per 1000” approach is thereby in the correct size, the problem left in the current approaches is “only” the rather high inefficiency of natural humification after plowing. The overall amount of easily available side stream biomass (≈10 Gt a^−1^ of waste biomass from industrial agriculture, only, e.g., 1 Gt per year sugarcane bagasse in Brazil), in combination with a synthetic, effective and decentral chemical humification processes allows us to consider as‐generated artificial humic matter as an effective tool for climate remediation, land rehabilitation and even terraforming.

This was already discussed in our previous publication.^44^ The picture of those days was however too simple: biochar and “hydrochar” are also carbonaceous, with similar composition, however with less functionality, and water and fertilizer binding for instance relies on ionic groups, redox buffering on phenolic groups, all that not contained in hard carbons. The product to synthesis is more than only a porous carbon. It must be as multifunctional as humins, the product of a long coevolution of plants and the rhyzosomal microbiome, as well as it has to interact and change the surface of the inorganic components of soil to create a hybrid structure.

Many of the challenges along such an “Apollo‐project” are already scientifically solved or currently addressed: the artificial humification process is stable and comparably cheap. The carbon yield even when based on mixed waste biomass is very high, and first tests in agriculture indicate very positive influence on soil structure, water and ion binding capacities and the final agricultural fertility and productivity.^45^ In our earlier work on hydrochar, we still found a slightly retarding influence on the soil mycorrhiza when the acidic hydrochar loadings in minced soil exceeded 10 wt%.^46^ The acid problem has been apparently fixed by moving from hydrochar to humic acid technology using the auto‐pH neutralizing chemistry, while of course osmotic pressure effects will always restrict application of a water binding material as soil additive toward too high concentrations. On the other hand, influence on the soil microbiome, the medium‐term stability of A‐HA in soil, as well as potential secondary solubilization issues and related health risks are still open questions and must be carefully analyzed in the next years.

We also like to talk about a possible new class of materials: Soil‐inspired materials. Soil scientists have described throughout their long endeavors many facets of humic acids. They bind water and ions and hydrophobic molecules, they are surface active and change and interact with minerals, they are active in pH and redox buffering and provide a stabilizing milieu for microbial and fungal activity, and so on. In industrial or materials chemistry, all these potentials are apparently either not known or not realized, although HA is presumably the biggest redox buffer on Earth. The purpose of this opinion article is to start a discussion on this “step beyond”: emulating humification by much faster and efficient chemical engineering processes can come up with artificial humic acids (A‐HA) or even “designer humic acids” with optimized wanted‐for properties, such as amphiphilicity. The use of such A‐HAs for agroindustrial and construction/industrial purposes might become indeed the first carbon‐negative product accessible on largest scales.

Beyond our scientific enthusiasm we have to realize: the billion‐ton scale is indeed very large and relies on political and agreed about international actions. The responsibility of science is however to show the feasibility of approaches and to start small. A‐HAs can be made from biomass waste, they are cheap and sustainable, and the authors yearly carbon footprint of 10 t (as a travelling scientists) could be compensated by terraforming of his garden, bringing higher fertility and added plant growth as a nonlinear, long lasting, sustainable effect to bind even more CO_2_. For nongardeners, we recall the options in replacing classical ion exchange resins, oil binding materials, redox batteries, environmental remediation, or concrete flux additives, all large‐scale applications in high demand for a carbon negative product.

## Conflict of Interest

The authors declare no conflict of interest.
